# Psychiatric morbidity and dietary habits during COVID-19 pandemic: a cross-sectional study among Egyptian Youth (14–24 years)

**DOI:** 10.1186/s43045-021-00085-w

**Published:** 2021-02-01

**Authors:** Roa Gamal Alamrawy, Noha Fadl, Asmaa Khaled

**Affiliations:** 1 Mamoura Psychiatric Hospital, General Secretariat of Mental Health and Addiction Treatment, El Nabawy El Mohandes Street, Mamoura, Alexandria, 21912 Egypt; 2 Family Health Department, High Institute of Public Health, Alexandria University, Alexandria, Egypt; 3 Community Health Nursing Department, Faculty of Nursing, Alexandria University, Alexandria, Egypt

**Keywords:** Psychiatric morbidity, Depression, Anxiety, Insomnia, Diet, COVID-19, Youth, Mental Health, Coping, Eating habits

## Abstract

**Background:**

The coronavirus disease 2019 (COVID-19) is influencing all segments of society, including youth. Although the physical complaints in the time of COVID-19 are broadly-studied, a paucity of research targeted psychological ones on the precious youth population. This study aimed to describe the real-time state of Egyptian youth’s psychiatric morbidity, dietary changes, and coping methods during this pandemic and explore probable factors influencing them. A descriptive cross-sectional study was conducted using an online survey among 447 Egyptian participants aged 14–24 years. Sociodemographic data, dietary habits, and coping methods during COVID-19 were collected. The Arabic versions of the Patient Health Questionnaire (PHQ-9), the Generalized Anxiety Disorder Scale (GAD-7), and the Insomnia Severity Index (ISI) were used to assess depression, anxiety and insomnia, respectively.

**Results:**

Overall, 80.5%, 74.0%, and 73.8% of the participants had different grades of depression, anxiety, and insomnia symptoms. 37.4% gained weight. Emotional and night eating emerged as new habits during the pandemic among 17.9% and 29.3% of the participants, respectively. Each of depression, anxiety, and insomnia was significantly associated with each other, female gender and having a COVID-19 infected relative. Adolescents had significantly higher scores of depression and anxiety. Those with a history of physical illness had significantly higher scores of anxiety and insomnia. Bodyweight and dietary changes were significantly associated with depression, anxiety, and insomnia. Participants reported various positive and negative coping methods.

**Conclusion:**

Psychiatric morbidity and dietary changes are evident in young people during COVID-19 pandemic. Psychological well-being and dietary habits are important but often overlooked components of youth well-being especially in challenging times. Depression, anxiety, and insomnia were almost always present and dietary changes were significantly associated with the presence of them.

## Background

Coronavirus disease (COVID-19) has become a potent destroyer of human health and the global economy in the twenty-first century and may be considered the most extraordinary global public health emergency nowadays. The World Health Organization (WHO) announced spreading of coronavirus rapidly across the globe, starting from China and becoming a pandemic by 11 March 2020. It has spread to 222 countries, affected over than 85 million people worldwide and killed over one million and half [1]. All continents announced confirmed cases of COVID-19. In Egypt, the first confirmed case was announced on 14 February 2020 [2].

Coronavirus disease (COVID-19) affects all segments of society worldwide. Despite that young people are less affected clinically by COVID-19 than adults, they are indirectly impacted by the pandemic [3]. Such a pandemic influenced the future of the young generation, the world’s most valuable asset.

Although recently published evidence heavily outlined the physical complaints in the time of COVID-19, a paucity of research targeted psychological ones [4]. In this context, daily stressful events, prolonged home confinement, financial worries, violence, and over usage of the internet are reasons that could affect the mental health of youth during this critical period [5]. In addition, most activities that usually occupy youth’s lives, such as schooling, social, and extracurricular activities with mates, have been disrupted. Putting all together, these will have broad-ranging influences on youth development and their mental health depending on their vulnerability and coping abilities in times of crisis [6]. These interruptions are expected to worsen or trigger mental illness, including anxiety, depression, and/or stress-related symptoms.

Psychological state of adults in Egypt has been investigated in the time of COVID-19 [7]. However, to date, no detailed research has been carried out on psychiatric morbidity and eating habits in young people facing the pandemic. Hence, the current study aims to describe the real-time state of Egyptian youth’s psychiatric morbidity, dietary habits, and coping methods during this pandemic and explore probable factors influencing them. It is an attempt to help in preserving the psychological well-being of the community.

## Methods

### Study design and study population

A descriptive cross-sectional study was performed using an online survey. Egyptian adolescents and young adults of both genders aged between 14 and 24 were reached through online advertisements on social media channels. Participants with a history of mental illness and debilitating physical disease were excluded. The survey was administered from 2 July to 23 July 2020.

Based on a prevalence rate of 58.0 % for depression symptoms among Egyptian youth with a mean age of 20.18 ± 1.50 years [8], using 5% accepted degree of precision, α of 0.05 and power of 95%, the minimum required sample size was 375 participants. The sample size increased up to 447 participants. The sample size was calculated using EPI-Info 2002 software [9].

### Measurement

A data collection sheet was constructed to collect sociodemographic data, including age, gender, residence, and medical history of chronic physical illness. Additionally, some questions related to COVID-19 infection, such as financial loss due to the COVID-19 pandemic and whether any of the family members got infected by COVID-19, were included.

Based on a systematic review [10], questions related to dietary changes during the COVID-19 pandemic were added, such as bodyweight changes, the number of meals/day, changes of dietary habits, emotional eating, and night eating in addition to consumption of the beverage, caffeinated and energy drinks. Emotional eating was defined as the propensity to over-eat as a direct response to negative emotions such as anxiety, fear, or stress. Night eating habit was described as intake of at least 25% of one’s total daily calories at night.

Coping methods among the participants were investigated by inquiry about the strategies used during the COVID-19 pandemic. These questions were conceptualized based on review of the literature [11–13]. Coping strategies were assessed by a question: “What do you do when the current pandemic burdens you?” 12 variables were used, each with 2 responses (no, yes). Coping methods were divided into 2 groups; positive (e.g., increase self-awareness regarding COVID-19, communication with other and seeking support, engagement in a hobby, exercise, and pray) and negative group (e.g., denial, tendency to stay alone, being aggressive and antisocial, smoking, consumption of hypnotics, consumption of caffeine, and substance abuse).

Depression was evaluated using the Arabic version of the Patient Health Questionnaire (PHQ-9) [14]. The PHQ-9 involves nine items representing the criterion symptoms for major depressive disorder by The Diagnostic and Statistical Manual of Mental Disorders, Fifth Edition (DSM-5) [15]. Participants were asked how many times they had been bothered by each symptom over the past 2 weeks, with response choices of not at all, several days, more than half the days, and nearly every day, scored as 0, 1, 2, and 3, respectively. Participants were categorized into four groups according to their scores, mild (5–9), moderate (10–14), moderately severe (15–19), and severe (20–27) [14].

For assessing anxiety symptoms, the Arabic version of the Generalized Anxiety Disorder Scale (GAD-7) was used [16]. The GAD-7 comprises 7 items with the same response options as PHQ-9. Although GAD-7 was originally developed as an indicator of generalized anxiety disorder, the GAD-7 operating characteristics are mostly as good for the other common anxiety disorders (e.g., panic disorder, social anxiety disorder, and post-traumatic stress disorder). GAD-7 scores were graded as 5–9 for mild, 10–14 for moderate, and 15–21 for severe [16] .

The validity and reliability of the Arabic versions of PHQ-9 and GAD-7 have been demonstrated among Saudi university students. Cronbach’s alpha value for the internal consistency reliability was 0.857 and 0.763 for the Arabic versions of PHQ-9 and GAD-7, respectively [17].

Insomnia was evaluated using The Arabic version of the Insomnia Severity Index (ISI) [18]. ISI assesses insomnia depending on criteria by the International Classification of Sleep Disorders [19]. The ISI has seven items to evaluate the perceived severity of sleep initiating difficulties, staying asleep, and early morning awakenings, satisfaction with current sleeping patterns, intervention on daily functioning, and noticeability of impairment attributed to the sleep problem, as well as the degree of distress or anxiety caused by sleep problems. The scores for symptoms severity were 8–14 for mild, 15–21 for moderate, and 22–28 for severe insomnia associated with significant daytime dysfunction [18]. The translated ISI showed adequate reliability and validity among the Arab population. Cronbach’s alpha coefficient was 0.84 [20].

### Statistical analysis

In order to explain the features of the study participants, frequency distribution and descriptive statistics (mean ± standard deviation) were used. Bivariate analysis was conducted using the chi-square test to evaluate the participants in the sample for the occurrence or absence of symptoms of depression, anxiety and insomnia. The exact Fisher test was used when chi-square test was not applicable. Depression, anxiety, and insomnia symptoms were represented in the bivariate analysis as dichotomous variables (no, yes). Statistical testing was conducted using statistical package for social science (SPSS version 21.0) [21]. Two-tailed *p* value < 0.05 was considered statistically significant.

### Ethics approval and consent to participate

Approval from the Ethics’ Committee of High Institute of Public Health, Alexandria University, was obtained before conducting the current study. After explaining the study’s purpose, all participants were provided with informed written online consent for participation and publication. For those who were less than 16 years old, informed written parental consent was obtained. The online survey was conducted anonymously to ensure the confidentiality of data. This work has been carried out in accordance with The Code of Ethics of the World Medical Association (Declaration of Helsinki) on Human participants.

## Results

Participants were between 14 and 24 years old *(n* = 447) with mean age of 20.72 ± 1.92 years, 21.3% were adolescents between 14 and 19 years old (*n* = 95). Out of them, 70.2% were females and three-quarters (74.9%) were urban residents. On the one hand, 9.4% reported having a family member infected with COVID-19. On the other hand, approximately two-thirds of their families suffered from financial loss due to COVID-19 (61.7%) (Table 1).

**Table 1:** Characteristics of the study participants (*n* = 447)

Characteristics	***n (%)***
**Gender**	
Male	133 (29.8)
Female	314 (70.2)
**Age, years** (mean ± SD = 20.72 ± 1.92)	
14–19	95 (21.3)
20–24	352 (78.7)
**Residence**	
Urban	335 (74.9)
Rural	112 (25.1)
**Medical history of chronic physical illness**	
No	427 (95.5)
Yes	20 (4.5)
**Did any member of your family get infected with COVID-19?**	
No	405 (90.6)
Yes	42 (9.4)
**Financial loss due to COVID-19**	
No	171 (38.3)
Yes	276 (61.7)
**Depression** (mean ± SD = 11.21 ± 6.58)	
None—minimal	87 (19.5)
Mild	106 (23.7)
Moderate	115 (25.7)
Moderately severe	76 (17.0)
Severe	63 (14.1)
**Anxiety** (mean ± SD = 9.31 ± 6.30)	
None—minimal	116 (26.0)
Mild	127 (28.4)
Moderate	104 (23.3)
Severe	100 (22.4)
**Insomnia** (mean ± SD = 12.367 ± 6.41)	
No clinically significant insomnia	117 (26.2)
Mild to moderate insomnia	161 (36.0)
Moderate insomnia	133 (29.8)
Severe insomnia associated with significant impairments of daytime functioning	36 (8.1)
Coping methods **	
**Positive methods**	
Raise self-awareness regarding COVID-19	403 (90.2)
Praying	374 (83.7)
Communication with other and seeking support	329 (73.6)
Engagement in a hobby	225 (50.3)
Exercise	179 (40.0)
**Negative methods**	
Tendency to stay alone	322 (72.0)
Being aggressive and antisocial	52 (11.6)
Denial	69 (15.4)
Smoking	36 (8.1)
Consumption of hypnotics	22 (4.9)
Substance abuse	6 (1.3)

*COVID-19* coronavirus disease 2019

^**^ Multiple responses

A minority of the participants (4.5%) reported history of chronic physical illness. The participants experienced different grades of depression (80.5%), anxiety (74%), and insomnia (73.8%). The most common positive coping methods practiced during COVID-19 pandemic were raising self-awareness regarding COVID-19, praying and communicating with others. Meanwhile, tendency to stay alone was the most common negative coping method (Table 1).

Regarding dietary changes during COVID-19, 37.4% gained weight while 20% reported weight loss. Around half of the sample (49.9%) had changes in their number of daily meals. Meanwhile, quarter of the sample (25.1%) reported increased consumption of healthy food, while 24.2% had increase in consumption of junk food. Emotional and night eating emerged as new habits during the pandemic among 17.9% and 29.3% of the participants, respectively. A change in consumption of carbonated beverages was reported in 49% while 50.3% had changes in their consumption of caffeinated and energy drinks Table 2.

**Table 2:** Dietary changes among the study participants during COVID-19 pandemic (*n* = 447)

Dietary changes	***n (%***
**Body weight change**	
No change	190 (42.5)
Weight loss	90 (20.1)
Weight gain	167 (37.4)
**Number of meals/day**	
No change	224 (50.1)
Decreased	120 (26.8)
Increased	103 (23.0)
**Changes of dietary habits**	
No change	152 (34.0)
Poor appetite	75 (16.8)
Increased consumption of junk food	108 (24.2)
Increased consumption of healthy food	112 (25.1)
**Emotional eating**	
Not a habit at all	251 (56.2)
A habit before COVID-19	116 (26.0)
A new habit during COVID-19	80 (17.9)
**Night eating**	
Not a habit at all	157 (35.1)
A habit before COVID-19	159 (35.6)
A new habit during COVID-19	131 (29.3)
**Consumption of carbonated beverage**	
No change	228 (51.0)
Decreased	131 (29.3)
Increased	88 (19.7)
**Consumption of caffeinated and energy drinks**	
No change	222 (49.7)
Decreased	96 (21.5)
Increased	129 (28.9)

*COVID-19* coronavirus disease 2019

Depression symptoms were significantly associated with female gender (*P* < 0.001), adolescent age-group (*P* = 0.002), having a family member infected with COVID-19 (*P* = 0.034), anxiety, and insomnia symptoms (*P* < 0.001). Similarly, female gender (*P* < 0.001), adolescence (*P =* 0.002), a history of physical illness (*P* = 0.029), having a family member infected with COVID-19 (*P* = 0.029), depression and insomnia symptoms (*P* < 0.001) were significantly associated with anxiety. Meanwhile, insomnia symptoms were significantly higher among females (*P* = 0.016), those with a history of physical illness (*P =* 0.028), had a family member infected with COVID-19 (*P* = 0.027), and presence of depression and anxiety (*P* < 0.001) (Table 3).

**Table 3:** Characteristics of the study participants by depression, anxiety, and insomnia symptoms (*n* = 447)

	Depression, *n* (%)		Anxiety, *n* (%)		Insomnia, *n* (%)	
	No 87 (19.5)	Yes 360 (80.5)	No 116 (26.0)	Yes 331 (74.0)	No 117 (26.2)	Yes 330 (73.8)
**Gender**						
Male	50 (37.6)	83 (62.4)	59 (44.4)	74 (55.6)	45 (33.8)	88 (66.2)
Female	37 (11.8)	277 (88.2)	57 (18.2)	257 (81.8)	72 (22.9)	242 (77.1)
* P value*	< 0.001 *		< 0.001 *		< 0.016 *	
**Age, years**						
14–19	8 (8.4)	87 (91.6)	16 (16.8)	79 (83.2)	20 (21.1)	75 (78.9)
20–24	79 (22.4)	273 (77.6)	100 (28.4)	252 (71.6)	97 (27.6)	255 (72.4)
* P value*	0.002 *		0.022 *		0.201	
**Residence**						
Urban	65 (19.4)	270 (80.6)	85 (25.4)	250 (74.6)	86 (25.7)	249 (74.3)
Rural	22 (19.6)	90 (80.4)	31 (27.7)	81 (72.3)	31 (27.7)	81 (72.3)
* P value*	0.956		0.630		0.676	
**Medical history of physical illness**						
No	86 (20.1)	341 (79.9)	115 (26.9)	312 (73.1)	116 (27.2)	311 (72.8)
Yes	1 (5.0)	19 (95.0)	1 (5.0)	19 (95.0)	1 (5.0)	19 (95.0)
* P value*	0.145		0.029 *		< 0.028 *	
**Did your family get infected with COVID-19?**						
No	84 (20.7)	321 (79.3)	111 (27.4)	294 (72.6)	112 (27.7)	293 (72.3)
Yes	3 (7.1)	39 (92.9)	5 (11.9)	37 (88.1)	5 (11.9)	37 (88.1)
* P value*	0.034 *		0.029 *		< 0.027 *	
**Financial loss due to COVID-19**						
No	31 (18.1)	140 (81.9)	50 (29.2)	121 (70.8)	49 (28.7)	122 (71.3)
Yes	56 (20.3)	220 (79.7)	66 (23.9)	210 (76.1)	68 (24.6)	208 (75.4)
* P value*	0.575		0.212		< 0.348	
**Insomnia**						
No	67 (57.3)	50 (42.7)	74 (63.2)	43 (36.8)	–	–
Yes	20 (6.1)	310 (93.9)	42 (12.7)	288 (87.3)	–	–
* P value*	< 0.001 *		< 0.001 *		–	
**Anxiety**						
No	79 (68.1)	37 (31.9)	–	–	74 (63.8)	42 (36.2)
Yes	8 (2.4)	323 (97.6)	–	–	43 (13.0)	288 (87.0)
* P value*	< 0.001 *		–		< 0.001 *	
**Depression**						
No	–	–	79 (90.8)	8 (9.2)	67 (77.0)	20 (23.0)
Yes	–	–	37 (10.3)	323 (89.7)	50 (13.9)	310 (86.1)
* P value*	–		< 0.001 *		< 0.001 *	

*COVID-19* coronavirus disease 2019

^*^P < 0.05

Bodyweight changes are significantly associated with each of depression, anxiety, and insomnia (*P* < 0.001). Dietary changes in form of number of daily meals, changes of dietary habits, practicing emotional and night eating recently, increased consumption of carbonated beverage, and increased consumption of caffeinated and energy drinks were significantly associated with existence of depression, anxiety, and insomnia symptoms (Table 4).

**Table 4:** Association between dietary changes during COVID-19 and depression, anxiety, and insomnia symptoms among the participants (*n* = 447)

	Depression, *n (%)*		Anxiety, *n (%)*		Insomnia, *n (%)*	
	No 87 (19.5)	Yes 360 (80.5)	No 116 (26.0)	Yes 331 (74.0)	No 117 (26.2)	Yes 330 (73.8)
**Bodyweight change**						
No change	66 (34.7)	124 (65.3)	82 (43.2)	108 (56.8)	77 (40.5)	113 (59.5)
Weight loss	7 (7.8)	83 (92.2)	10 (11.1)	80 (88.9)	9 (10.0)	81 (90.0)
Weight gain	14 (8.4)	153 (91.6)	24 (14.4)	143 (85.6)	31 (18.6)	136 (81.4)
* P value*	< 0.001 *		< 0.001 *		< 0.001 *	
**No. of meals/ day**						
No change	69 (30.8)	155 (69.2)	91 (40.6)	133 (59.4)	88 (39.3)	136 (60.7)
Decreased	6 (5.0)	114 (95.0)	10 (8.3)	110 (91.7)	9 (7.5)	111 (92.5)
Increased	12 (11.7)	91 (88.3)	15 (14.6)	88 (85.4)	20 (19.4)	83 (80.6)
* P value*	< 0.001 *		< 0.001 *		< 0.001 *	
**Changes of dietary habits**						
No change	33 (21.7)	119 (78.3)	50 (32.9)	102 (67.1)	56 (36.8)	96 (63.2)
Loss of/poor appetite	3 (4.0)	72 (96.0)	6 (8.0)	69 (92.0)	3 (4.0)	72 (96.0)
Increased consumption of junk food	8 (7.4)	100 (92.6)	11 (10.2)	97 (89.8)	16 (14.8)	92 (85.2)
Increased consumption of healthy food	43 (38.4)	69 (61.6)	49 (43.8)	63 (56.2)	42 (37.5)	70 (62.5)
* P value*	< 0.001 *		< 0.001 *		< 0.001 *	
**Emotional eating**						
Not a habit at all	70 (27.9)	181 (72.1)	88 (35.1)	163 (64.9)	80 (31.9)	171 (68.1)
A habit before COVID-19	12 (10.3)	104 (89.7)	21 (18.1)	95 (81.9)	24 (20.7)	92 (79.3)
A new habit during COVID-19	5 (6.3)	75 (93.8)	7 (8.8)	73 (91.3)	13 (16.2)	67 (83.8)
* P value*	< 0.001 *		< 0.001 *		< 0.006 *	
**Night eating**						
Not a habit at all	52 (33.1)	105 (66.9)	59 (37.6)	98 (62.4)	68 (43.3)	89 (56.7)
A habit before COVID-19	21 (13.2)	138 (86.8)	39 (24.5)	120 (75.5)	27 (17.0)	132 (83.0)
A new habit during COVID-19	14 (10.7)	117 (89.3)	18 (13.7)	113 (86.3)	22 (16.8)	109 (83.2%)
* P value*	< 0.001 *		< 0.001 *		< 0.001 *	
**Consumption of carbonated beverage**						
No change	60 (26.3)	168 (73.7)	70 (30.7)	158 (69.3)	72 (31.6)	156 (68.4)
Decreased	22 (16.8)	109 (83.2)	34 (26.0)	97 (74.0)	34 (26.0)	97 (74.0)
Increased	5 (5.7)	83 (94.3)	12 (13.6)	76 (86.4)	11 (12.5)	77 (87.5)
* P value*	< 0.001 *		0.008 *		< 0.003 *	
**Consumption of caffeinated and energy drinks**						
No change	60 (27.0)	162 (73.0)	70 (31.5)	152 (68.5)	76 (34.2)	146 (65.8)
Decreased	17 (17.7)	79 (82.3)	23 (24.0)	73 (76.0)	21 (21.9)	75 (78.1)
Increased	10 (7.8)	119 (92.2)	23 (17.8)	106 (82.2)	20 (15.5)	109 (84.5)
* P value*	< 0.001 *		0.016 *		< 0.001 *	

*COVID-19* coronavirus disease 2019

^*^P < 0.05

## Discussion

The COVID-19 pandemic may negatively influence mental health as a consequence of the distinctive blend of the public health crisis, social isolation and economic crises [22]. Measures to contain the virus have reshaped many facets of daily life, such as income, employment, schooling, and social interactions. Considering the direct impact of the virus and related consequences, researchers anticipated an increase in anxiety, depression, and their aftermath, including suicide [23]. Results of the current study revealed that depression and anxiety were almost always present with different severities. This high percentage agrees with a Chinese survey among adolescents aged 12–18; as symptoms of depression and anxiety during the COVID-19 outbreak were highly prevalent [24]. Similarly, in a study in Spain [25], during the first weeks of COVID-19 confinement, large numbers of students experienced moderate to extremely severe scores of anxiety and depression.

In comparing the results of the present study to other studies during COVID-19 pandemic, the current sample PHQ-9 mean score for depression (11.21 ± 6.58) was higher than that of Lin’s et al. [26], who studied 5641 Chinese individuals across the different age groups, as the average PHQ-9 score of their participants was 6.10 ± 5.97. Also, in the same study, the anxiety scores assessed using GAD-7 had an average score of 4.97 ± 5.25 [26], while the current sample GAD-7 mean score was (9.31 ± 6.30) which is higher.

Furthermore, with a mean ISI score of 12.36 ± 6.41, the prevalence of clinical insomnia was 73.8**%**. This is much higher than reported before in a Chinese study; the average ISI score was 5.93 ± 5.88 and the prevalence of clinical insomnia was 20.05% [26]. While in other studies, insomnia was identified in 23% of 11,835 young adults [27]. Youth experienced distress, lack of energy, irregular naps, more screen time, and unrestricted online social media. A well-recognized feature of Egyptian sleep practice is biphasic sleep distributed in afternoon and late night bouts. Also, most of Egyptians eat the main meal of the day around mid-afternoon, and then nap in the late afternoon. In the evening, activity picks up again. The night bout of sleep begins sometime after midnight and ends after dawn [28]. These disruptive effects of social disorganization can affect bedtime, sleep quality, and sleep/wake patterns constancy directly or collectively [29].

Evidence found that depression and mental states among Arab adolescents are constructed and expressed differently than among adolescents in other cultures [30]. Culture may play a role in differences in frequency of disorders in different groups [31]. Also, socio-cultural factors may also play a part in how distress is expressed [32]. Hence, the differences across different population studies may emerge.

The present study showed significant associations between symptoms of depression, anxiety, and insomnia. Previous studies shown that symptoms of anxiety and depression are risk factors for insomnia and are associated and cyclically related [27, 33]. The relationship between sleep disturbance and depression is complex from the level of shared neurobiology to manifestation in clinical symptoms. Sleep duration and depressive symptoms appear to be related concurrently in a curvilinear manner [34]. Given the higher mean score of PHQ-9 in the current study, this might contribute to the higher prevalence of insomnia in comparison to other studies. Studies found that almost 75% of children and adolescents with depression manifested sleep disturbances, with over 50% experiencing insomnia [35]. Social distance and isolation that follows long-term lockdowns can be a risk factor for anxiety and mood disorders [36]. Variations in sleep during the COVID-19 pandemic may exacerbate or even contribute to psychopathology. Poor sleep can have a detrimental effect, leading to heightened vulnerability to mood and anxiety disorders [37].

An accumulating body of evidence indicated that females are at higher risk of depression and anxiety symptoms [24, 38], sleep problems [39], and negative psychological impact in general [7]. These results also matched the findings of this study, where female gender has significant associations with depression, anxiety, and insomnia. On the other hand, a study by Xie et al. reported no effect of gender [40].

Notably, youth between 14 and 19 had more severe scores on depression, anxiety but not insomnia, when compared to the older youth. Other studies revealed that younger persons reported higher psychological impacts [7, 26]. These results are contradicting with a study Zhou et al. that found prevalence of symptoms of insomnia was higher among senior high school and college students [27]. The inverse correlation between respondents' age and the prevalence of depressed mood, anxiety, and insomnia was recorded in another study [41]. In the studied population, age has no significant association with insomnia. The timing and amount of sleep are largely determined by direct and interactive effects of gender, age, and sleeping arrangements [28].

It appears that the residence may give an idea about the circumstances in which the person lives and hence on his social conduct [42]. The result of the current study reveals that residence had no effect on association with psychopathology. This was in contrast to studies in China that found that depression and anxiety symptoms in cities were lower than in rural areas [24, 43]. A Greek study also found that people in urban areas were more vulnerable to sleep problems [39], while an Egyptian study found rural residency had a negative impact on mental health during COVID-19 [7]. This might be implied by the fact of urbanization of the countryside and ruralization of cities is taking place [44]. Considerably, the association of psychopathology and residence may change based on the population studied.

The mind and mental health are influenced by the health of the body. This is observed in the present study where young people with chronic physical illness had significantly higher anxiety and insomnia scores but not depression. El-Zoghby et al. reported that those with chronic illnesses are more susceptible to psychological distress [7]. Furthermore, some of them may perceive themselves with poor health and more liable to get diseased [45, 46]. Even after controlling for confounders, strong cross-effects between physical and mental health are still valid [47].

Moreover, those who had a family member infected with COVID-19 had more significant distress in the form of depression, anxiety, and insomnia. These findings are corresponding to Cao et al., who found that having relatives or acquaintances with COVID-19 infection were risk factors for anxiety among Chinese undergraduate students [43]. Besides, all family members may have their COVID-19-related fears. Taken together, this response can lead to significant psychological distress for all family members, especially youth.

Economic recessions as during pandemic are accompanying with increased mental health problems for youth [22]. Family income has a significant impact on young people response to life stressors such as the COVID-19 crisis [48]. In a study among 7143 college students, family income stability was a protective factor against anxiety in the era of COVID-19 [43]. However, the results of the current study revealed that financial loss affected neither mood nor sleep. Although, low socioeconomic status is generally associated with high psychiatric morbidity, the nature of this association is not clear-cut [49]. Part of the difficulty is in disentangling other factors and their interactions, which are beyond the scope of current study.

Concerning changes in bodyweight during COVID-19 lockdown, 37.4% of the sample reported weight gain, 20.1% reported weight loss, while around half of the sample reported change in their number of daily meals. These are comparable to a study in Poland, in which around 30% and over 18% experienced weight gain and loss respectively, while over 43.0% and nearly 52% reported eating and snacking more [50]. Consistently, a study [51] on 3533 Italians found the sense of satiety and hunger altered for more than 50% of their population; 17.8% of them had less appetite, comparable to 16.8% of the sample. Phillipou et al. provided further support and reported increased restricting and binge eating behaviors among Australian population [52]. Notably, isolation leads to an increased sedentary behaviors and more physical inactivity which are expected to affect weight [53]. The excessive food exposure caused by the only freedom permitted was grocery shopping, which prompted people who were least successful in managing their diet to amplify the association between food consumption and feelings [41].

Furthermore, the current study is in line with an extensive body of evidence indicated that food consumption, eating patterns, food type, number of main meals, emotional and night, in addition to beverages consumption (caffeinated and carbonated), all were affected during confinement [54]. In addition to bodyweight, all described dietary changes have significant associations with depression, anxiety and insomnia. Previous studies reported increased scores of emotional and night eating in response to external stimuli with PHQ-9 and GAD-7 scores [55].

Pandemic-related anxiety may increase the difficulty of patients in managing their eating habits [56]. From another point of view, the consumption of palatable foods may have positive and reinforcing implications. The stress response can be sensibly controlled through its soothing effect [57]. However, the current study pointed out that around one-quarter of the sample improved their diet in consuming a more healthy diet and nearly the third decreased their intake of carbonated beverages. These findings were also reported by Di Renzo et al. but only 16.7% of their population improved their behaviors in the form of a decrease in consumption of snacks, savory food, carbonated, and sugary drinks [51].

Participants of both sexes reported various positive and negative coping strategies. In stress situation, coping mechanisms are used to control, mitigate, or tolerate stress and stressors such as those during COVID-19. A study involved 16 countries found that the most utilized coping strategies during COVID-19 were watching television for entertainment, social networking, listening to music, sleeping, performing household chores like cleaning and washing, eating well, and clearing/finishing piled-up work [58]. While the strategies used the most by participants of the current study were raising self-awareness regarding COVID-19, praying, communication with others, staying alone, and engagement in a hobby.

This study sheds important light on youth’s psychiatric morbidity and dietary habits during the COVID-19 pandemic. Up to our knowledge, this study is one of the first studies to address such matters in Egypt and the Middle East. However, there is no study without limitations. The cross-sectional design of the current study limits the possibility of establishing temporality. Additionally, the psychological status was assessed using a self-administered online survey. A systematic psychiatric evaluation through interviews of the participants would give a better assessment. Dietary habits and coping methods were assessed using designed questionnaires. Although these questionnaires were developed after a careful review of literature, the tools were new and could add to the limitations.

## Conclusion

Psychiatric morbidity and dietary changes are evident in young people during COVID-19 pandemic. Psychological well-being and dietary habits are important but often overlooked components of youth well-being especially in challenging times. Results of the current study highlighted the presence of depression, anxiety, insomnia during the COVID-19 pandemic. This study also gave an idea about dietary changes during the pandemic and various coping methods. These findings suggest that, as the pandemic continues to evolve, it is important to continue the monitoring of young people well-being. More controlled studies are needed to assess the causality between stress during the pandemic, mental health impacts and dietary changes. In future studies, assessment of dietary habits and coping methods using validated questionnaires would be of an added value. Putting it all together, timely action could mitigate the current negative state and improve mental health and well-being. Developing a strategy targeting fulfilling these unmet needs is just one way to help our youth deal with the uncertain coming days.

## Data Availability

Data is available upon request.

## Abbreviations

COVID-19: Coronavirus disease 2019
DSM: Diagnostic and Statistical Manual of Mental Disorders
GAD-7: Generalized anxiety disorder scale
ISI: Insomnia Severity Index
PHQ-9: Patient Health Questionnaire
WHO: The World Health Organization
